# Synergistic Effect of Maleated Natural Rubber and Modified Palm Stearin as Dual Compatibilizers in Composites based on Natural Rubber and Halloysite Nanotubes

**DOI:** 10.3390/polym12040766

**Published:** 2020-04-01

**Authors:** Nabil Hayeemasae, Zareedan Sensem, Indra Surya, Kannika Sahakaro, Hanafi Ismail

**Affiliations:** 1 Department of Rubber Technology and Polymer Science, Faculty of Science and Technology, Prince of Songkla University, Pattani Campus, Pattani 94000, Thailand; zareedan@gmail.com (Z.S.); kannika.sah@psu.ac.th (K.S.); 2 Department of Chemical Engineering, Faculty of Engineering, Universitas Sumatera Utara, Medan 20155, Sumatera Utara, Indonesia; isurya@usu.ac.id; 3 School of Materials and Mineral Resources Engineering, Engineering Campus, Universiti Sains Malaysia, Nibong Tebal 14300, Penang, Malaysia; ihanafi@usm.my

**Keywords:** natural rubber, maleated natural rubber, palm stearin, halloysite nanotubes

## Abstract

The performance of rubber composite relies on the compatibility between rubber and filler. This is specifically of concern when preparing composites with very different polarities of the rubber matrix and the filler. However, a suitable compatibilizer can mediate the interactions. In this study, composites of natural rubber (NR) with halloysite nanotubes (HNT) were prepared with maleated natural rubber (MNR) and modified palm stearin (MPS) as dual compatibilizers. The MPS dose ranged within 0.5–1.5 phr, while the MNR dose was fixed at 10 phr in all formulations. It was found that the mixed MNR/MPS significantly enhanced modulus, tensile strength, and tear strength of the composites. The improvements were mainly due to improved rubber-HNT interactions arising from hydrogen bonds formed in the presence of these two compatibilizers. This was clearly verified by observing the Payne effect. Apart from that, the MPS also acted as a plasticizer to provide improved dispersion of HNT. It was clearly demonstrated that MNR and MPS as dual compatibilizers improved rubber-HNT interactions and reduced filler-filler interactions, which then improved tensile and tear strengths, as well as dynamical properties. Therefore, the mix of MNR and MPS had a great potential to compatibilize non-polar rubber with HNT filler.

## 1. Introduction

Enhanced properties of rubber can be obtained by adding a small amount of nanofillers. This technique has drawn considerable attention during the last decades [1,2,3]. The improvements of physical and other related properties of rubber depend on several factors, such as the filler aspect ratio, filler’s compatibility, the degree of dispersion, and the alignment of the particulates. Halloysite nanotubes (HNT) are a type of nanofiller that has been recently tested in many types of matrix [4,5,6,7]. This is because of the very special characteristics of this material formed by surface weathering of aluminosilicate minerals and composed of aluminum, silicon, hydrogen, and oxygen. Due to the unique surface chemistry of HNT, it is not compatible with non-polar rubbers, such as natural rubber (NR). Scientists have been trying to address this drawback by several approaches to improve their compatibility. These include using silane coupling agents [8], adjusting the preparation methods [9], and using compatibilizers [10]. 

The silane coupling agent has been widely used and reported for the last decades. The use of silane is known but considered to be expensive and requires a high mixing temperature to obtain effective silanization. The chemistry behind the enhancement of the composites has also been very well studied. In the meantime, adjusting the processing methods has also been focused on the preparation of rubber composites. As, for example, Varghese and Karger-Kocsis [9] prepared natural rubber/layered silicates through latex compounding method. They found out that the latex route was promising, but it might not be of great practical relevance in comparison to the melt-compounding route. Besides, the acting shear forces strongly favor the dispersion of filler and is a strong argument for melt compounding with rubbers. Thus, searching for an alternative and effective compatibilizer for rubber/HNT composite is of great interest. A compatibilizer tends to affect the overall structure of a composite. However, most common compatibilizers are synthetic chemicals, and there has been less focus on the utilization of natural-based compatibilizers. In this study, two types of compatibilizer were used to modify the compatibility of NR with HNT: one being a modified natural rubber, and another being modified palm stearin. 

As for the modified natural rubber, the compatibility of NR and HNT can also be improved by some functional groups. Pasbakhsh et al. [11] prepared maleic anhydride (MA) grafted ethylene propylene diene rubber (EPDM) or EPDM-g-MA, to increase the compatibility of EPDM and HNT. It was obvious that the use of EPDM-g-MA reduced HNT agglomeration and hence improved the HNT dispersion. This was attributed to interactions between the hydroxyl groups on HNT surfaces and succinic anhydride groups of the EPDM-g-MA. Similar approaches can be found in the literature [12,13]. They have also proposed possible interactions between hydroxyl groups of the paper sludge and succinic anhydride groups. 

Despite adding modified natural rubber as a compatibilizer, further improvements in material properties can be obtained by introducing another compatibilizer. Palm stearin is an interesting material that has been useful in natural rubber compounds. Palm stearin is derived from extensive processing in the palm oil factory. It is fractionated from the refinery processing of crude palm oil [12], and its value is lower compared to the main product (palm olein). Due to its waxy character, this material can act as a plasticizer and improve the processability of rubber. Apart from the physical appearance of palm stearin, the chemical substances available in palm stearin are also interesting. It consists of highly saturated fats and triglycerides [13]. These components react with amines to produce unique chemical substances called fatty acid amides [14,15]. From the structural point of view, the interactions between NR and HNT could be improved by modified palm stearin. Recently, Surya et al. [14] reported on the use of modified palm stearin in a NR/silica composite, and it was found that the modified palm stearin gave shorter scorch and cure times, whereby the torque difference, tensile modulus, tensile strength, hardness, and crosslink density increased up to 5 phr doses of modified palm stearin. Similar observations have been reported for carbon black filled NR composites, showing that the mechanical properties have been improved by modified palm stearin [16]. 

The aim of this study was to use modified palm stearin (MPS) as a mixed compatibilizer with maleated natural rubber (MNR) for NR/HNT composites. Based on the chemical structures of both MNR and MPS, they are anticipated to provide better compatibility, especially at the outer layers of HNT (silanol and/or siloxane groups). To date, no reports have been published of detailed investigations concerning the use of dual compatibilizers from MNR and MPS to improve the mechanical and morphological evolution of NR/HNT composites. This study would bring a scientific perspective on the role of MNR and MPS as dual compatibilizers for NR/HNT composites and provide detailed information for manufacturing rubber products with HNT filler.

## 2. Materials and Methods

### 2.1. Materials

The main NR matrix used in this experiment was STR 5L, which was manufactured by Chalong Latex Industry Co., Ltd. (Songkhla, Thailand). The HNT was manufactured by Imerys Ceramics Limited, (Kerikeri, New Zealand). HNT consists of the following components: SiO_2_ (49.5 wt %), Al_2_O_3_ (35.5 wt %), Fe_2_O_3_ (0.29 wt %), TiO_2_ (0.09 wt %), as well as CaO, MgO, K_2_O, and Na_2_O as traces. The palm stearin was fractionated and supplied by Chumporn Palm Oil Industry PCL. (Chumporn, Thailand). The curing activators ZnO and stearic acid were purchased from Global Chemical Co., Ltd. (Samut Prakan, Thailand) and Imperial Chemical Co., Ltd. (Bangkok, Thailand), respectively. N-cyclohexyl-2-benzothiazole sulfenamide (CBS), used as an accelerator, was supplied by Flexsys America L.P. (Creve Coeur, MO, USA), and sulfur, used as vulcanizing agent, was bought from Siam Chemical Co., Ltd. (Samut Prakan, Thailand). Other chemicals involved in the preparation of MNR and MPS, such as maleic anhydride, sodium methoxide, diethanolamine, diethyl ether, and saturated sodium chloride, were purchased from Sigma-Aldrich (Thailand) Co., Ltd. (Bangkok, Thailand).

### 2.2. Synthesis of MPS

**Figure 1:**
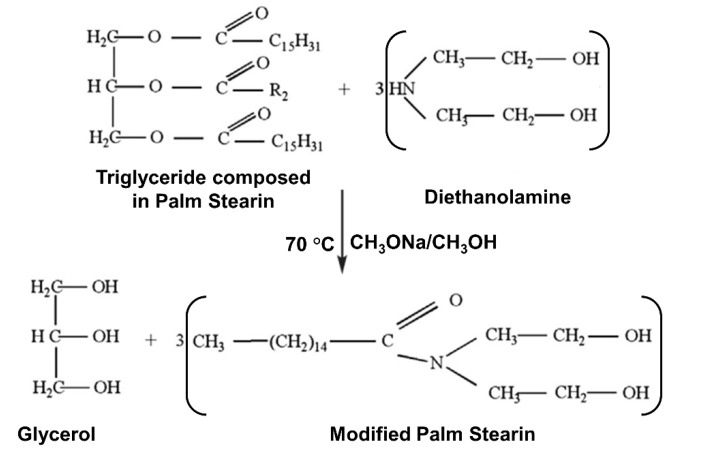
The chemical reaction between triglyceride-based palm stearin and diethanolamine (modified from Surya et al. [14]).

The synthesis of MPS followed the procedure described by Surya et al. [14]. This was done in a reaction kettle fitted with a stirrer at atmospheric pressure. The methanol was initially mixed together with sodium methoxide while stirring. The mixture of palm stearin and diethanolamine was then added to the mixture while stirring. The reaction was carried out at 70 °C for 5 h. The mixture was later extracted and washed with diethyl ether and saturated sodium chloride solution. Finally, the crude MPS was purified with anhydrous sodium sulfate and concentrated in a rotary evaporator prior to use. The MPS was stored in a desiccator prior to characterization with Fourier transform infrared spectroscopy (FTIR) to assess changes in functionalities. The reaction to make MPS is shown in Figure 1. The MPS appeared as a cream-colored wax and was used as a compatibilizer to improve the compatibility in NR/HNT composites.

### 2.3. Synthesis of MPS

Grafting of MA onto NR was done by mixing the NR with 4 phr of MA in a Brabender Plasticorder at 145 °C at a rotor speed of 60 rpm under a normal atmosphere. The mixing lasted for 10 min. The resulting rubber was purified by reprecipitation. This was done just for the purpose of characterization by FTIR. The resulting MNR was then purified to confirm the grafting of MA onto NR. This was carried out by dissolving the rubber sample in toluene at room temperature for 24 h and then at 60 °C for 2 h. The soluble part was collected and precipitated in acetone. The sample was dried in a vacuum oven at 40 °C for 24 h. The purified MNR was finally characterized by the FTIR spectrum.

### 2.4. Preparation of Composites based on NR and HNT

Table 1 depicts the main ingredients to prepare the rubber composites, and MNR was used in all the modified compounds. Here, reference compound is denoted for the composite without compatibilizer. MPS 0 phr is the composite with only MNR as compatibilizer, MPS 0.5–1.5 phr are the composites with MNR/MPS as dual compatibilizers at MPS contents from 0.5–1.5 phr respectively. The entire amounts of additives were mixed in a Brabender (Plastograph® EC Plus, Mixer W50EHT 3Z, Brabender ® GmbH & Co. KG, Duisburg, Germany), and, just after dumping, the compounds were passed through a two-roll mill to avoid overheating prior to determining curing characteristics. The compounds were then compressed into certain shapes using a hydraulic hot press, with the vulcanizing times obtained by using a moving-die rheometer (MDR) as described later.

### 2.5. Attenuated Total Reflection-Fourier Transform Infrared Spectroscopy (ATR-FTIR)

The FTIR spectra of MNR were analyzed using a Bruker FTIR spectrometer (Tensor 27, Bruker Optik GmbH, Baden-Württemberg, Germany) with a smart durable single bounce diamond in the ATR cell. Each spectrum was recorded in transmission mode after 32 scans per spectrum, with 4 cm^−1^ resolution from 4000 to 400 cm^−1^.

### 2.6. Determination of Curing Characteristics

A moving die rheometer or MDR (Rheoline, Mini MDR Lite, Prescott instruments Ltd., Gloucestershire, UK) was utilized to determine the curing characteristics of the composites. The tests were carried out according to ASTM D5289 at 150 °C. The data recorded were torques, scorch time (t_s1_), and curing time (t_c90_).

### 2.7. Measurement of Mechanical Properties

The samples were cut into a dumbbell shape, according to ASTM D412. The tensile tests were conducted using a universal tensile machine (Tinius Olsen, H10KS, Tinius Olsen TMC, Horsham, PA, USA) at a cross-head speed of 500 mm/min. This was done to determine 100% modulus, 300% modulus, tensile strength, and elongation at break. Further, tear strengths of the composites were also tested using the same machine by following ASTM D624 with a cross-head speed of 500 mm/min. The tear strength recorded was the average of five repeated tests for each compound.

### 2.8. Dynamic Properties

The dynamic properties of the NR/HNT composites in the presence of dual compatibilizers were studied using a Rubber Process Analyzer model D-RPA 3000 (MonTech Werkstoffprüfmaschinen GmbH, Buchen, Germany). The composite sample was cured at 150 °C for the curing time obtained from Rheoline Mini MDR Lite (Prescott Instruments Ltd., Gloucestershire, UK). Then, the sample was cooled down to 60 °C and deformed at 10 Hz frequency, varying the strain in the range from 0.5 to 100%. The raw outputs storage modulus (*G’*) and damping characteristics (tan δ) were recorded, and the rubber-filler interactions in the composites were assessed by the Payne effect. The Payne effect was calculated as follows.

Payne effect = *G’_i_* – *G’_f_* (1)

where *G’_i_* is *G’* at 0.5% strain, and *G’_f_* is the *G’* at 100% strain. A larger Payne effect indicates lesser rubber-filler interactions.

### 2.9. Scanning Electron Microscopy

Fractured samples from tensile testing were used to assess the microdefects. Imaging was carried out using a scanning electron microscope (FEI Quanta 400 ESEM, Thermo Fisher Scientific, Waltham, MA, USA) to obtain information on the dispersion of the HNT filler throughout the NR matrix, in both the absence and presence of MNR/MPS as compatibilizers. The fractured pieces were sputter-coated with gold–palladium to eliminate electrostatic charge buildup during imaging.

## 3. Results and Discussion

### 3.1. Functionalities of Maleated Natural Rubber

**Figure 2:**
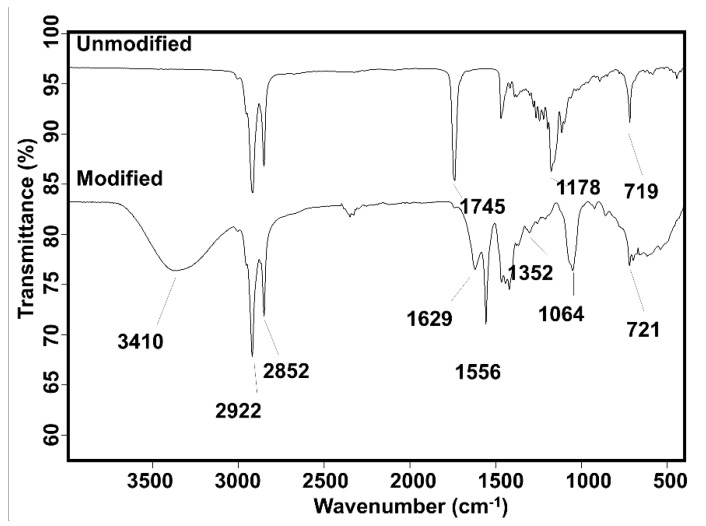
FTIR spectra of unmodified and modified palm stearin.

The typical infrared spectra of unmodified and modified palm stearin are shown in Figure 2. The wavenumbers and their respective assignments are summarized in Table 2. Similar bands of C–H stretch were detected at wavenumbers 2922 and 2852 cm^−1^, and CH_2_ rocking bands appeared at 719 and 721cm^−1^ associated with long alkyl chains in the fatty acids; these were observed in both unmodified and modified palm stearin. Further evidence of a methyl group (CH_3_) attached to a carbon atom was shown by the umbrella mode at 1352 cm^−1^ [17]. The distinct bands observed in the modified palm stearin distinguishing it from unmodified palm stearin are also shown in Figure 2. These included the strong band for O–H stretch at 3410 cm^−1^, the C=O stretch at 1629 and 1556 cm^−1^, and the amide C–N stretch at 1064 cm^−1^, respectively. The spectrum clearly indicated the functional groups present in the proposed MPS chemical structure seen in Figure 1.

### 3.2. Functionalities of Maleated Natural Rubber

**Figure 3:**
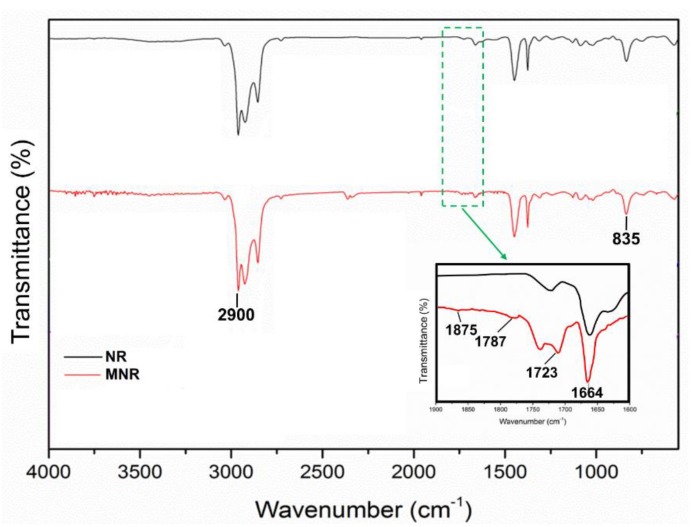
FTIR spectra of MNR prepared at various MA contents. NR, natural rubber; MNR, maleated natural rubber; MA, maleic anhydride.

FTIR spectra of MNR at various MA contents are shown in Figure 3, while the peak assignments are listed in Table 3. A broad and intense band at 1787 cm^−1^ and a weak absorption band at 1875 cm^−1^ were observed. These bands could be assigned to the successfully grafted anhydride and were due to symmetric (strong) and asymmetric (weak) C=O stretching vibrations of succinic anhydride rings, respectively. The observed bands were clearly indicating succinic anhydride groups grafted onto NR molecules. Moreover, there was an important peak captured at wavenumber 1723 cm^−1^ due to the formation of carbonyl groups of opened ring structure succinic anhydride. The peaks seen in this study were quite similar to previous results in the literature [18,19].

### 3.3. Cure Characteristics

**Figure 4:**
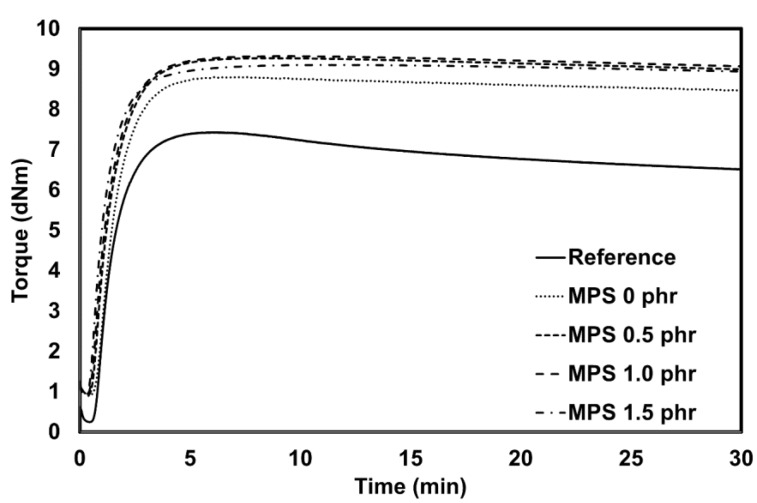
Curing curves of NR/HNT composites in the presence of MNR/MPS as a dual compatibilizer.MPS, modified palm stearin.

The curing curves of the NR/HNT composites with and without MNR/MPS as dual compatibilizers are illustrated in Figure 4, and the results are also summarized in Table 4. The maximum torque (M_H_) decreased on adding MPS but then increased again with further increases in the MPS dose, showing that the MPS played an important role in improving the compatibility of the NR/HNT composites. A similar trend was observed for the torque difference (M_H_ – M_L_), which is the difference between maximum torque (M_H_) and minimum torque (M_L_). This value is known to indicate the degree of cross-linking and/or interactions within the composite system [20], so this could indicate that the compatibility of NR with HNT was significantly enhanced when MPS was added to the composite. The amide groups in MPS also shortened the scorch (t_s1_) and cure times (t_c90_) of the composites. As mentioned before, MPS was synthesized from palm stearin and diethanolamine, which made MPS an alkaline substance. This increased the pH of the rubber compounds, which tended to increase the cure rate. Any chemical substance that makes the rubber compound more alkaline will increase the cure rate, while acidity tends to retard the reactivity of accelerators [21]. It was, therefore, expected that the amine in MPS could accelerate the cure rate of the composites.

### 3.4. Mechanical Properties

**Figure 5:**
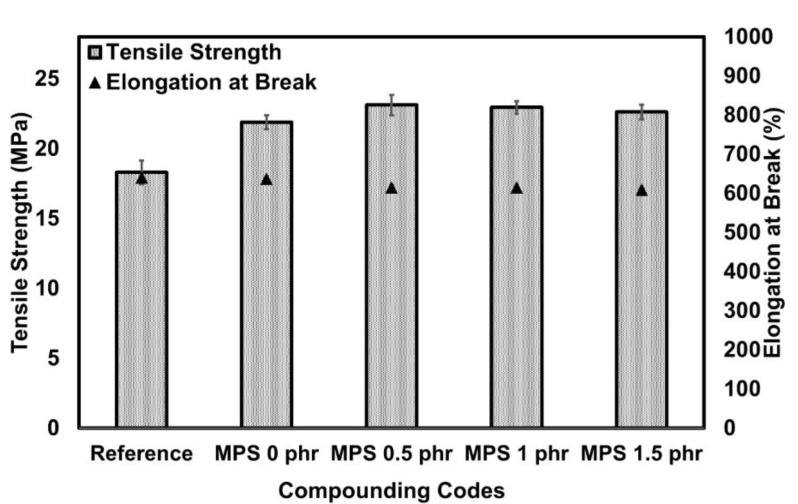
Tensile strength and elongation at break of NR/HNT composites in the presence of MNR/MPS as dual compatibilizers.

**Scheme 1:**
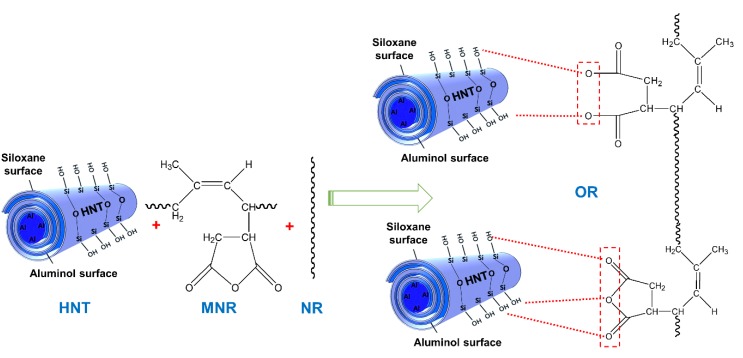
Possible interactions between NR and HNT in the presence of MNR as compatibilizer.

**Scheme 2:**
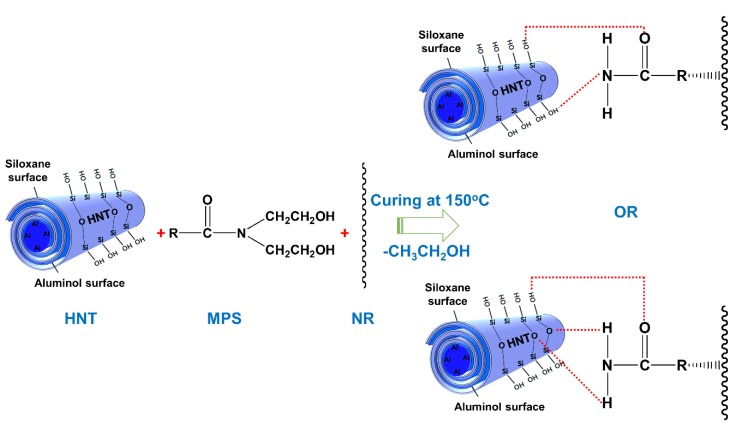
Possible interactions between NR and HNT in the presence of MPS as compatibilizer.

Tensile strength and elongation at break of NR/HNT composites with and without MNR/MPS as a dual compatibilizer are shown in Figure 5. The tensile strength increased upon the incorporation of MPS. Improved tensile strength was attributed to the MPS itself, which has great potential to mediate interactions between NR and HNT. The mixed compatibilizers obviously influenced the tensile strength of this composite, as could be seen from the tensile strengths of the composites without (reference) and with dual compatibilizers (MPS 0–1.5 phr). Here, the interaction between NR and HNT was discussed for each compatibilizer used. Scheme 1 shows the proposed interaction obtained by the action of MNR, with two possible interactions in the composite either through the opened ring and/or cyclic structures. Grafting of the succinic anhydride groups onto NR molecules of the MNR increased the polarity of rubber and made it compatible with HNT. Pasbakshs et al. [11] also proposed the same interactions between hydroxyl groups of HNT and succinic anhydride groups of EPDM-g-MA. As for the MPS, the interactions were between amide groups of MPS and siloxane groups at the outer surfaces of HNT (see Scheme 2), and these reactions increased the reinforcing efficiency in the NR/HNT composites.

**Figure 6:**
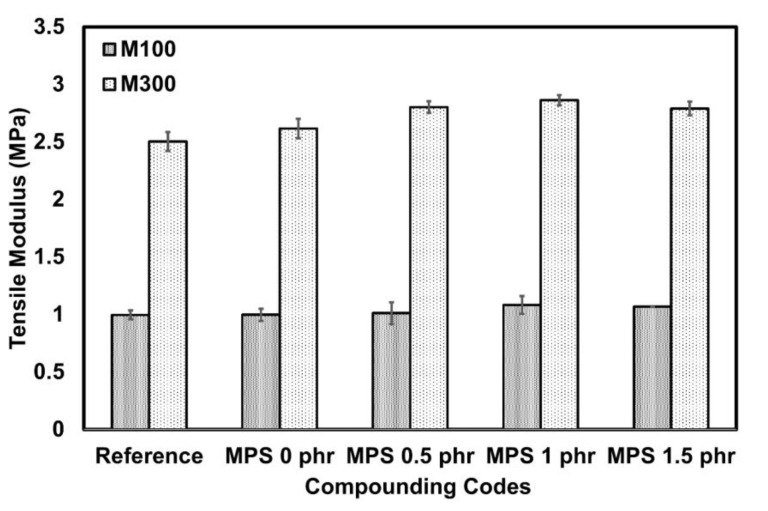
Tensile modulus of NR/HNT composites in the presence of MNR/MPS as a dual compatibilizer.

**Figure 7:**
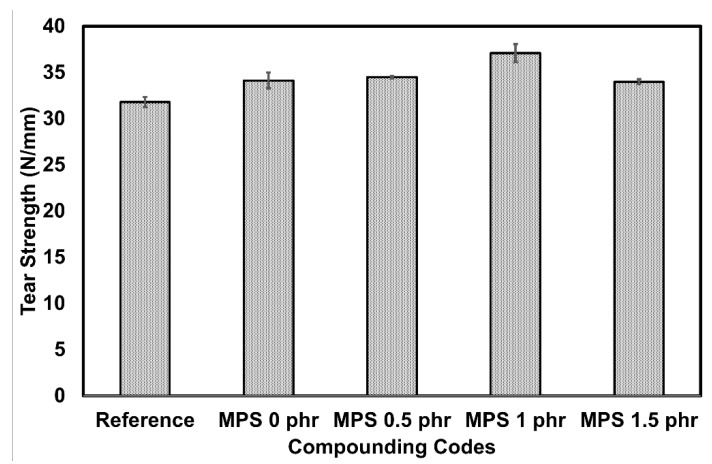
Tear strength of NR/HNT composites in the presence of MNR/MPS as dual compatibilizers.

The strong interactions of NR and HNT could be confirmed from the stresses at 100% and 300% strain (see Figure 6). It could be seen that the stresses at 100% and 300% elongations (M100 and M300) increased with MPS dose. As more MPS was added to the rubber, more interactions took place, resulting in stiffer and harder composites. This was in good agreement with the M_H_ and M_H_ – M_L_ reported in the preceding section. In addition to this, the tear strength also showed remarkable improvement upon the inclusion of MPS, as could be seen in Figure 7. The improved tear strength was simply due to the strong interactions between NR and HNT, as well as the ability of MPS to improve the dispersion of HNT filler in the NR matrix.

### 3.5. Dynamic Properties

**Figure 8:**
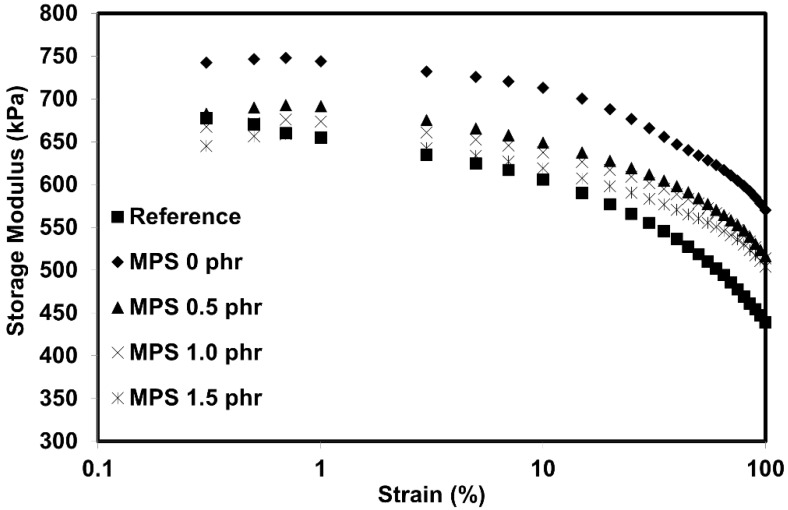
The storage modulus (*G’*) of NR/HNT composites in the presence of MNR/MPS as a dual compatibilizer.

**Figure 9:**
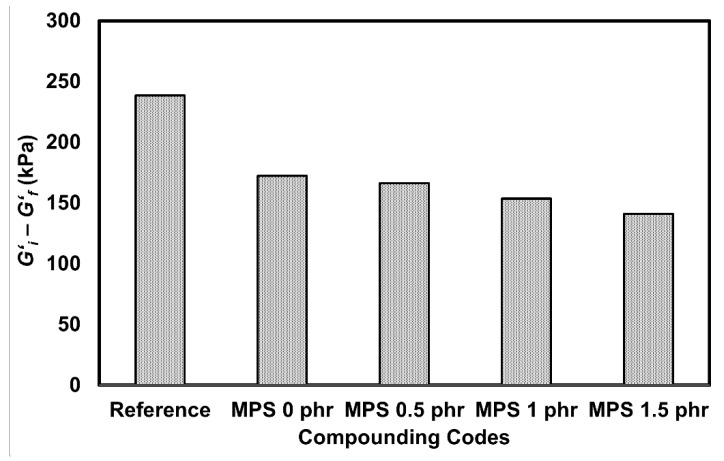
Payne effect (*G’_i_* – *G’_f_*) of NR/HNT composites in the presence of MNR/MPS as a dual compatibilizer.

The dynamic properties of the composites were determined with a Rubber Process Analyzer to investigate the storage modulus and the Payne effect. Figure 8 and Figure 9 show the storage modulus (*G’*) and the Payne effect (*G’_i_* – *G’_f_*) of NR/HNT composites. It could be seen that the storage modulus of gum NR was constant in the low strain region but slightly decreased when the strain exceeded 50%. This is a common phenomenon for viscoelastic materials and is due to the molecular stability of rubber. In addition to that, the Payne effect was estimated as the difference between storage modulus at low and high strain amplitudes [22,23]. The level of the Payne effect for the composite without MNR/MPS as a dual compatibilizer was found to be 238.65 kPa, and this decreased to 172.40 kPa for the composite with solely MNR. This was a good indication that the interactions between NR and HNT were improved by MNR. Moreover, the Payne effect was reduced, on introducing MPS as a second compatibilizer, to 166.30, 153.61, and 141.02 kPa, respectively, for MPS loadings of 0.5, 1, and 1.5 phr. The lower Payne effect indicates lesser filler-filler interactions [24]. 

**Figure 10:**
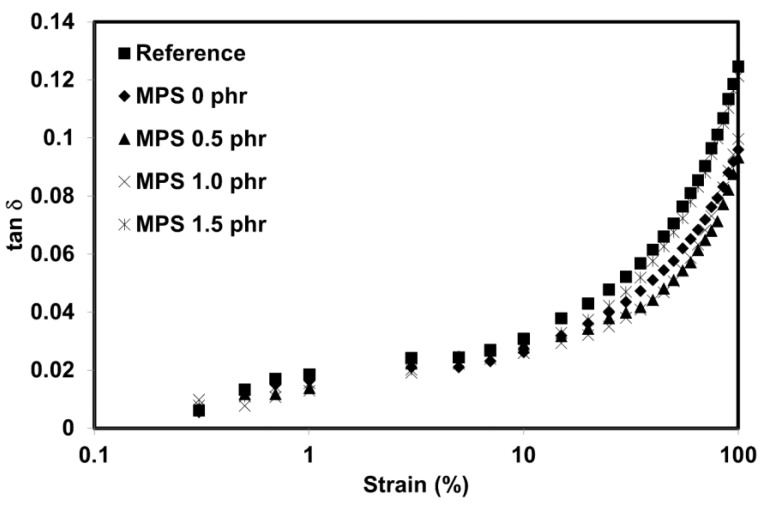
Damping characteristic (tan δ) of NR/HNT composites in the presence of MNR/MPS as a dual compatibilizer.

Dependence of the damping characteristic (tan δ) on strain is shown in Figure 10. It is obvious that the composites exhibited low damping characteristics after the addition of MPS, indicating a considerable degree of molecular mobility. This was simply due to the better interactions between NR and HNT after adding MNR/MPS as a dual compatibilizer. The improved compatibility of rubber-HNT increased the interfacial adhesion and resulted in improved elastic properties of the vulcanizates.

### 3.6. Scanning Electron Microscopy

**Figure 11:**
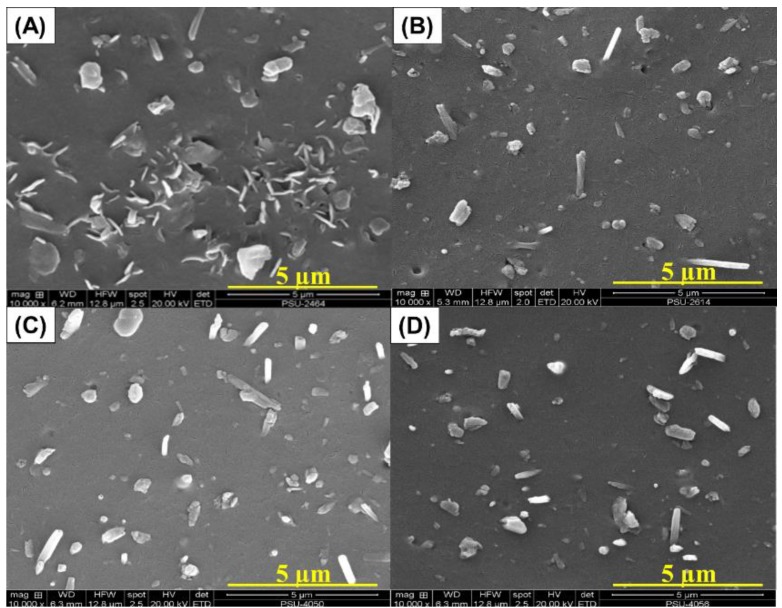
SEM images of tensile fractured surfaces of NR/HNT composites in the presence of MNR/MPS as a dual compatibilizer: Reference (**A**), MPS 0 phr (**B**), MPS 0.5 phr (**C**), and MPS 1.5 phr (**D**), all at 10,000× magnification.

Figure 11 shows SEM micrographs of the tensile fractured surfaces of the NR/HNT composites with and without MNR/MPS as a dual compatibilizer at 10k× magnification. From Figure 11A, agglomeration of HNT is seen at a fractured surface of the NR/HNT composite without MNR and MPS. Upon the inclusion of MNR (see Figure 11B), agglomeration was no longer seen due to the compatibilizing effect of MNR. Furthermore, the dispersion of HNT was enhanced by MPS, and the homogeneity of the composite was significantly improved, especially at 1.5 phr dose level (see Figure 11C,D). The improved dispersion of HNT was clearly responsible for the improved tensile strength and tear strength, with more energy needed to break the sample. Better dispersion of HNT throughout the matrix increased the stress at any given strain. Similar observations were previously reported for microfractured surfaces of filled NR composites in the presence of a compatibilizer [25,26].

## 4. Conclusions

The overall properties of composites based on NR and HNT were clearly improved by adding MNR/MPS as a dual compatibilizer. Both MNR and MPS had special functional groups that could form hydrogen bonds with the hydroxyl groups on HNT surfaces. MPS alone could also promote the dispersion of HNT filler in the NR matrix due to its waxy character. The filler-matrix interactions improved the mechanical properties, such as tensile strength, modulus, and tear strength of the composites, and this mechanism was corroborated by the decreased Payne effect observed in dynamic measurements. It was clearly demonstrated in this present work that the use of MNR and MPS improved the rubber-HNT interactions and reduced filler-filler interactions, providing great benefits to the mechanical and dynamical properties. This finding contributed to understanding the roles of MNR and MPS as dual compatibilizers for NR/HNT composites and could be a source of useful information for manufacturing such composites. To some extent, this could lead to modifying processing methods without requiring commercial compatibilizers that are costly and complicate the process.

## Figures and Tables

**Table 1:** Compounding ingredients used to prepare composites.

Ingredient	Compounding Code and Amounts in phr				
	Reference	MPS 0 phr	MPS 0.5 phr	MPS 1.0 phr	MPS 1.5 phr
NR	100	90	90	90	90
MNR *	-	10	10	10	10
ZnO	5	5	5	5	5
Stearic acid	1	1	1	1	1
CBS	2	2	2	2	2
Sulfur	2	2	2	2	2
HNT	10	10	10	10	10
MPS	-	-	0.5	1	1.5

Remark: * MNR prepared at 4 phr of maleic anhydride. MPS, modified palm stearin; NR, natural rubber; MNR, maleated natural rubber; HNT, halloysite nanotubes; CBS, N-cyclohexyl-2-benzothiazole sulfenamide.

**Table 2:** The peaks and their assignments in spectra for unmodified and modified palm stearin.

Wavenumber (cm^-1^)	Assignment
3410	O–H stretch
2922, 2852	C–H stretch
1745	C=O stretch in ester
1629, 1556	C=O stretch in a modified structure
1352	CH_3_ umbrella mode
1064	C–N stretch
719/721	CH_2_ rocking

**Table 3:** The peaks and their assignments in spectra for NR and MNR.

Wavenumber cm^−1^	Assignment
2900	C–H stretch of NR
1875	C=O stretch of succinic anhydride (weak)
1787	C=O stretch of polymeric anhydride (weak)
1723	C=O stretch of a carbonyl group
1664	C=C stretch of NR
835	C–H out of plane bend of NR

**Table 4:** Curing characteristics of NR/HNT composites in the presence of MNR/MPS as a dual compatibilizer.

Compound Code	M_H_ (dN.m)	M_H_ − M_L_ (dN.m)	t_S1_ (min)	t_c90_ (min)
Reference	7.43	7.18	0.84	2.81
MPS 0 phr	7.33	6.38	1.25	3.16
MPS 0.5 phr	9.27	8.35	0.7	2.84
MPS 1.0 phr	9.32	8.37	0.64	2.76
MPS 1.5 phr	9.1	8.2	0.56	2.43
